# Food Addiction in Individuals With Overweight and Obesity Undergoing a 12‐Week Long Tailored mHealth Weight Loss Intervention

**DOI:** 10.1002/erv.3196

**Published:** 2025-04-03

**Authors:** Magdalena Pape, Stephan Herpertz, Tanja Färber, Caroline Seiferth, Natalie Schoemann, Jörg Wolstein, Sabine Steins‐Loeber

**Affiliations:** ^1^ Department of Clinical Psychology and Psychotherapy University of Bamberg Bamberg Germany; ^2^ Department of Psychosomatic Medicine and Psychotherapy LWL‐University Hospital of the Ruhr‐University Bochum Bochum Germany; ^3^ Department of Pathopsychology University of Bamberg Bamberg Germany; ^4^ Department of Clinical Psychology and Psychotherapy Freie Universität Berlin Berlin Germany

**Keywords:** eating disorders, eating pathology, eating styles, food addiction, mHealth, weight loss

## Abstract

**Objective:**

Former studies indicated worse outcome for individuals with food addiction (FA) when participating in weight loss programs. Yet, the results may have been influenced by comorbid eating disorders and insufficient consideration of psychological aspects associated with FA.

**Methods:**

We report here a subgroup analysis on treatment success of participants with overweight/obesity and FA, but no comorbid eating disorders, compared to individuals with overweight/obesity but without FA and eating disorders taking part in a 12‐week long tailored mHealth weight loss intervention (IG) that addresses psychological aspects of overweight/obesity or a waitlist control condition (CG). Body‐mass‐index and eating styles were assessed at four time points: baseline, 3, 9 and 15 months after baseline.

**Results:**

Overall, the IG significantly reduced weight and improved eating styles. The efficacy of the intervention was higher in the IG + FA concerning long‐term weight loss and emotional eating.

**Conclusion:**

These findings suggest that individuals with FA, without comorbid eating disorders, benefit from a tailored mHealth weight loss intervention that focuses on psychological aspects of overweight/obesity. This underscores the presence of a FA subgroup among individuals with overweight and obesity, characterised by unique vulnerabilities and treatment responses, which should be further analysed. There is a need for specialised treatment of FA components in obesity and overweight.

**Trial Registration:**

ClinicalTrials.gov identifier: NCT04080193

**Summary**

- This study analysis food addiction in individuals with overweight/obesity but without comorbid eating disorders.
- A tailored mHealth intervention could improve emotional eating and reduce weight in individuals with food addiction.
- The consideration of food addiction in future studies might contribute to a better understanding of the obesity spectrum.

## 1 Introduction

In Germany, about 7.9% of the population fulfil the criteria of food addiction (FA) (Hauck et al. 2017). These individuals describe themselves as being addicted to specific highly processed (HP) foods (Schulte et al. 2015). HP foods are usually high in fat and contain refined sugars that can be absorbed quickly. The consumption can cause activations in the dopaminergic reward‐related neural systems, which are similar to those in other addictive disorders (Gearhardt et al. 2011; Pursey et al. 2024). Moreover, individuals with FA experience problems in controlling their eating behaviour, strong cravings for HP foods, continuation of the consumption despite negative consequences and repeated failures in quitting the problematic behaviour (Schulte et al. 2015). FA is associated with higher eating pathology in individuals seeking weight loss programs (WLPs) (Meule et al. 2015). Eating pathology in terms of dysfunctional eating styles influences weight loss and weight loss maintenance. For example, emotional eating (EE), that is eating caused by emotional states, contributes to overweight and obesity and is associated with the consumption of highly palatable foods (Konttinen et al. 2010; van Strien et al. 2013). Eating as a response to external food cues (external eating, EXE), for example the smell of foods, seem to impair weight maintenance after weight loss (Neumann et al. 2018; Van Strien et al. 1986; van Strien et al. 2009) and individuals with FA show high cue‐reactivity toward HP foods (Schulte et al. 2019). An improvement in dietary restraint eating (RE) is associated with greater weight loss undergoing WLPs (van Strien et al. 2009), yet individuals with FA often fail to quit consuming HP foods (Schulte et al. 2015).

FA is associated with higher body‐mass‐index (BMI) (Pursey et al. 2014). Prevalence rates of FA in individuals with overweight and obesity range between 17.2% and 25.0% (Hauck et al. 2017; Pursey et al. 2014). The prevalence of FA is even higher (47.2%–96.0%) in clinical samples of individuals with eating disorders (De Vries and Meule 2016; Gearhardt et al. 2014). Especially the presence of Binge‐eating‐disorder (BED) is associated with higher FA symptom severity (Penzenstadler et al. 2019). FA is currently not classified as a distinct disorder by the World Health Organization. There is still an ongoing debate on the existence of a FA construct, that is on the distinction to existing eating disorders (Fletcher and Kenny 2018; Gearhardt and Hebebrand 2021; Hebebrand and Gearhardt 2021). Lack of control over eating behaviour is a shared feature also observed in individuals with BED, resulting in binge eating episodes of huge amounts of food. As BED is not characterised by compensatory behaviours, it is often associated with overweight and obesity, too (McCuen‐Wurst et al. 2018). Some researchers therefore define FA as an ‘overeating disorder’ and more like a syndrome of other disorders, especially BED, rather than an isolated disorder (Hebebrand and Gearhardt 2021). However, despite the shared similarities, the criteria of FA cannot be transferred one‐to‐one to those of BED. For example, individuals with FA crave for specific HP foods. These cravings must not necessarily cause binge eating episodes, but might also result in ‘grazing’ specific foods throughout the day (Gearhardt and Hebebrand 2021). Independent from BED symptom severity, FA is associated with higher impulsivity and loss of control eating (Kalan et al. 2024). Whereas for individuals with BED, binge eating is caused by emotional distress and not exclusively directed toward HP foods. In an online survey of *N* = 502 individuals with overweight and obesity, 17% met FA criteria, 9% BED criteria and 10% FA and BED criteria (Ivezaj et al. 2016). However, the criteria were measured using self‐reporting questionnaires for both disorders.

The impact of FA on the efficacy of weight loss programs (WLPs) has been described heterogeneously, with most studies indicating worse outcomes for individuals with FA (De Almeida et al. 2022). Camacho‐Barcia et al. analysed *n* = 448 individuals (*M*_age_ = 65.25 years, SD = 4.63) with overweight or obesity (Camacho‐Barcia et al. 2021). They were randomly assigned to either an intervention group (IG) that received an intensive lifestyle intervention including a Mediterranean diet, physical activity promotion and behavioural support, or a control group (CG) that received a simple diet recommendation. Participants that fulfiled the criteria of FA at baseline (*N* = 26, 5.8%) reported higher BMI at baseline and were more likely to regain weight. In addition, other studies examining the impact of FA in participants undergoing weight loss lifestyle interventions reported that FA at baseline and higher FA symptom severity were associated with less weight loss and study failure (Fielding‐Singh et al. 2019; Gordon et al. 2020). On the other hand, a study of two different 6‐month lifestyle interventions in an outpatient setting, found no differences between participant with and without FA (Lent et al. 2014). However, none of the above‐mentioned studies systematically excluded other eating disorders. Since FA is highly comorbid with BED and BED is associated with less weight loss and higher drop‐out in WLPs (Forman et al. 2023), the results might have been influenced. Moreover, given the complexity of FA as a potential mental disorder, lifestyle interventions alone might not sufficiently target psychological parameters associated with FA, such as dysfunctional eating styles. The I‐GENDO app is a tailored mHealth intervention targeting psychological aspects associated with overweight and obesity (Pape et al. 2022). Prior analyses indicated that the intervention effectively improves eating pathology (Schoemann et al. 2024; Seiferth et al. 2023).

Taken together, individuals with FA seem to be a specifically burdened subgroup of individuals with overweight and obesity, suffering from high eating pathology and higher BMI. Prior studies indicate less weight loss and higher weight regain in individuals with FA undergoing lifestyle WLPs. Yet, it is not clear whether the results were influenced by comorbid eating disorders and if psychological aspects associated with FA were sufficiently addressed (De Almeida et al. 2022). We hypothesise that individuals with FA undergoing a 12‐week long tailored psychological mHealth intervention would reduce and sustain BMI. In addition, the impact of FA on dysfunctional eating styles will be analysed exploratively. The investigation may contribute to a better understanding of the obesity spectrum and improve future obesity treatment strategies.

## 2 Methods

### 2.1 Project

Data for this manuscript were retrieved from the I‐GENDO study a multicenter RCT designed to assess the efficacy of a tailored mHealth intervention aimed at reducing weight and improving eating styles. The overall results of the RCT have been previously published (Seiferth et al. 2023).

The study was conducted in accordance with the Declaration of Helsinki and approved by the Institutional Review Board of the Ruhr‐University Bochum (Nr. 18‐6415, 14 January 2019) and the ethical board of the University of Bamberg. All subjects were informed about the study and provided written informed consent.

### 2.2 Participants

Individuals were recruited between August 2019 and August 2020. Individuals interested in participation received a link to an online screening survey assessing eligibility criteria of the study (Table 1).

**TABLE 1:** Eligibility criteria of the I‐GENDO study.

Inclusion criteria	Exclusion criteria
Legal age (≥ 18 years)	Obesity class III (i.e., BMI > 39.9 kg/m^2^)
Obesity class I or II with subjectively experienced weight related impairment and a current intention to lose weight	Current (or within the last 12 months) involvement in a structured weight loss intervention Insulin‐dependent type 1 diabetes Previous or intended bariatric surgery Current psychotherapeutic treatment of weight related health problems
Overweight (i.e., BMI between 25 and 29.9 kg/m^2^) with weight related health problems and/or visceral adipose tissue and/or high psychosocial weight‐related distress with a current intention to lose weight	Weight‐enhancing drugs Drugs which promote weight loss (e.g., anti‐obesity drugs) Weight‐enhancing health problems which are not yet treated
	Cancerous disease within the last 5 years
	Current substance‐use disorders, major depression, psychosis, suicidal tendency or pregnancy Severe cognitive impairments Insufficient knowledge of the German language
	Binge‐eating‐disorder or bulimia nervosa

Individuals with comorbid eating disorders were excluded through a two‐step standardised procedure. Initially, suspected eating disorders were assessed using the Munich ED‐Quest (Fichter et al. 2015) in the online screening survey. Subjects flagged for potential eating disorders were subsequently diagnosed by experienced clinicians using the German version of the eating disorder examination (EDE) (Hilbert and Tuschen‐Caffier 2016). Individuals diagnosed with bulimia nervosa (BN) or Binge eating disorder (BED) as classified by the DSM‐5 criteria (American Psychiatric Association 2018) were excluded from participation. Eligible participants were invited to an in‐person appointment for baseline assessment.

The final sample consisted of 213 individuals (67.1% female) with obesity or overweight (*M*_BMI_ = 33.35 kg/m^2^, SD_BMI_ = 3.79 kg/m^2^). Ages ranged from 19 to 71 years (*M*_age_ = 46.45 years, SD_age_ = 12.13 years). Participants were randomly assigned to one of the study arms using block randomisation: intervention group (IG) (*n* = 116, female = 77) or control group (CG) (*n* = 97, female = 66). Computer‐generated lists were stratified by gender and study centre. Both groups completed questionnaires and reported their current weight (kg) and height (m) at four online assessments: baseline (T0), post‐treatment (T1, 3 months after baseline), follow‐up 1 (T2, 9 months after baseline) and follow‐up 2 (T3, 15 months after baseline). *N* = 183 of the 213 initial participants completed the T1 assessment (*n*_IG_ = 101, *n*_CG_ = 82), *N* = 161 participants completed the T2 assessment (*n*_IG_ = 93, *n*_CG_ = 68) and *N* = 152 participants completed the T3 assessment (*n*_IG_ = 90, *n*_CG_ = 62). A total of *N* = 143 participants (*n*_IG_ = 87, *n*_CG_ = 56) provided data at each of the four assessments, indicating a dropout rate of 32.9%. All data was stored on protected servers at the University of Bamberg.

### 2.3 Intervention

The I‐GENDO app is a 12‐week individualised and gender‐sensitive mHealth intervention that combines computer‐based and self‐tailoring features. The psychological intervention encompass five modules (stress management skills, emotion regulation skills, dealing with the consequences of overweight, inhibitory control/impulsive eating, and self‐efficacy) of which three were assigned as main modules to each participant. A detailed description of the development and process evaluation has been published elsewhere (Pape et al. 2022). Participants in the CG received no intervention during the study period. After completing T3, the I‐GENDO app was offered to the CG. The participants of the CG were aware that they would receive the app intervention after completing T3 (waiting‐group design).

### 2.4 Measures

Questions about sociodemographic data were included in the online questionnaire at baseline, whereas weight and height were reported at each of the four assessments. BMI was calculated by dividing the reported body weight in kilograms by height in metres squared (kg/m^2^).

#### 2.4.1 Food Addiction

FA over the past 12 months was measured using the German version of the revised Yale Food Addiction Scale (YFAS Scale 2.0) (Meule et al. 2017). The YFAS 2.0 consists of 35 items scored on an 8‐point scale ranging from ‘0' (never) to ‘7’ (every day). Based on 33 items, the 11 diagnostic criteria for substance‐use disorders in the DSM‐5 (e.g., craving, tolerance, or withdrawal) adapted to FA can be calculated. A symptom score (range: 0–11) reflects FA symptom severity. Additionally, two items assess clinically significant impairments or distress due to eating behaviour. FA is diagnosed when two or more symptoms are met (symptom score ≥ 2) along with clinically significant impairments or distress due to eating behaviour. The German version of the YFAS 2.0 was validated in a student sample and in a sample of patients with obesity and showed good psychometric properties (Meule et al. 2017). In the present sample, the YFAS 2.0 demonstrated excellent internal consistency (*α* = 0.91).

#### 2.4.2 Eating Styles

The German version of the Dutch Eating Behaviour Questionnaire (DEBQ, Nagl et al. 2016) was used to assess on three different eating styles: emotional eating (EE), restraint eating (RE) and external eating (EXE). The questionnaire consists of 30 items, scored on a 5‐point scale ranging from ‘1’ (never) to ‘5’ (very often). Higher mean scores on the subscales indicate a stronger expression of the corresponding eating style. The German version of the DEBQ was validated in a representative German sample and showed good psychometric properties (Nagl et al. 2016). In the present sample, the internal consistency of the subscales was acceptable for RE, *α* = 0.78, good for EXE, *α* = 0.84 and excellent for EE, *α* = 0.92.

### 2.5 Statistical Analysis

All analyses were conducted using IBM SPSS Statistics for Windows (Version 29.0, Armonk, NY: IBM Corp.) and Microsoft Excel (Version 16.0, Microsoft Corporation). Descriptive analyses included percentages and frequencies for categorical variables, and means and standard deviations for continuous variables. Baseline value comparisons between groups (IG + FA, IG − FA, CG + FA, CG − FA) were tested using chi‐square tests for categorical variables and analyses of variances (ANOVAs) for continuous variables. The Last Observation Carried Forward (LOCF) method was used to replace missing values of the outcome measures. To analyse the progress of BMI (within‐subject: BMI) over time (T0–T3), repeated measures mixed analysis of variance (rmANOVA) was conducted between groups (IG vs. CG) with and without FA (+FA vs. −FA) (between‐subject: IG + FA, IG − FA, CG + FA, CG − FA). The Greenhouse–Geisser adjustment was applied to correct for violations of sphericity. Bonferroni‐adjusted post hoc tests were performed to compare results between the four measurement points and between groups. In order to support the results of our primary analysis on the impact of FA on BMI, intention‐to‐treat (ITT) analyses was conducted with a linear multilevel regression model using maximum likelihood estimation. A two‐level model structure including a random intercept was applied. The model was adjusted for baseline BMI and the time × group × FA interaction term was added to the final model. Intraclass correlation coefficient (ICC) of the null model indicated that 91% of the variance was due to between‐person effects.

Secondary, rmANOVAs on the progress of eating styles (within‐subject: EE, RE, EXE) over time (T0–T3) were conducted between groups with and without FA (between‐subject: IG + FA, IG − FA, CG + FA, CG − FA).

## 3 Results

No significant group differences on sociodemographic values at baseline were found (Table 2). Table 3 displays descriptive data on BMI and eating styles progress over time (T0–T3).

**TABLE 2:** Sociodemographic data between groups at baseline.

	Overall (*N* = 213)	IG + FA (*n* = 19)	IG − FA (*n* = 97)	CG + FA (*n* = 13)	CG − FA (*n* = 84)	
Sex	*n*_*f*_ = 143 (67.1%)	*n*_*f*_ = 16 (84.2%)	*n*_*f*_ = 61 (62.3%)	*n*_*f*_ = 10 (76.9%)	*n*_*f*_ = 56 (66.7%)	*χ*(3) = 3.88, *p* ^e^ = 0.275
	*n*_*m*_ = 70 (32.9%)	*n*_*m*_ = 3 (15.8%)	*n*_*m*_ = 36 (37.7%)	*n*_*m*_ = 3 (23.1%)	*n*_*m*_ = 28 (33.3%)	
Age (years)	*M* = 46.45 SD = 12.13	*M* = 49.68 SD = 12.15	*M* = 46.80 SD = 11.56	*M* = 46.23 SD = 12.31	*M* = 45.33 SD = 12.78	*F*(3, 209) = 0.71 *p* ^e^ = 0.545
Marital status^a^	*n* = 170 (79.8%)	*n* = 16 (84.2%)	*n* = 75 (77.3%)	*n* = 10 (76.9%)	*n* = 69 (82.1%)	*χ*(3) = 0.95, *p* ^e^ = 0.813
Education^b^	*n* = 61 (27.8%)	*n* = 6 (31.6%)	*n* = 30 (30.9%)	*n* = 2 (15.4%)	*n* = 23 (27.4%)	*χ*(3) = 1.51, *p* ^e^ = 0.680
Employment^c^	*n* = 88 (41.3%)	*n* = 7 (36.8%)	*n* = 45 (46.4%)	*n* = 5 (38.5%)	*n* = 31 (36.9%)	*χ*(3) = 1.91, *p* ^e^ = 0.592
Nationality^d^	*n* = 211 (99.6%)	*n* = 19 (100.0%)	*n* = 97 (100.0%)	*n* = 12 (92.3%)	*n* = 83 (98.8%)	*χ*(3) = 7.53, *p* ^e^ = 0.057

Abbreviations: BMI = body mass index; CG = control group; FA = food addiction; IG = intervention group; YFAS = Yale Food Addiction Scale.

^a^ Number of participants living in a relationship.

^b^ Number of participants with a university degree.

^c^ Number of participants currently in employment.

^d^ Number of participants with German nationality.

^e^ Bonferroni‐adjusted *p* values.

**TABLE 3:** Descriptive data of BMI and eating styles at baseline, 3, 9 and 15 months assessment separated for groups.

	Baseline				3 months				9 months				15 months			
	IG + FA	IG − FA	CG + FA	CG − FA	IG + FA	IG − FA	CG + FA	CG − FA	IG + FA	IG − FA	CG + FA	CG − FA	IG + FA	IG − FA	CG + FA	CG − FA
	Mean (SD)				Mean (SD)				Mean (SD)				Mean (SD)			
BMI	34.26 (3.32)	33.44 (3.88)	34.79 (3.32)	32.80 (3.80)	33.36 (4.09)	33.03 (4.11)	34.85 (3.43)	32.63 (3.93)	32.72 (4.44)	33.31 (4.37)	35.05 (3.66)	32.57 (4.03)	32.69 (4.51)	33.22 (4.46)	34.55 (4.10)	32.53 (4.04)
EE	3.5 (0.88)	2.93 (1.08)	3.99 (0.76)	2.96 (0.92)	3.28 (1.19)	2.87 (0.99)	3.79 (0.88)	3.02 (0.93)	3.03 (1.00)	2.97 (0.96)	3.70 (0.90)	3.07 (1.00)	3.06 (1.18)	2.98 (1.02)	3.92 (0.82)	3.04 (0.95)
RE	2.92 (0.58)	2.64 (0.58)	2.64 (0.45)	2.82 (0.60)	3.27 (0.72)	3.00 (0.58)	2.62 (0.52)	2.93 (0.66)	3.33 (0.80)	2.89 (0.65)	2.59 (0.69)	2.93 (0.64)	4.14 (0.88)	2.83 (0.66)	2.48 (0.66)	2.86 (0.71)
EXE	3.53 (0.67)	3.46 (0.69)	3.82 (0.71)	3.41 (0.60)	3.40 (0.86)	3.30 (0.69)	3.62 (0.90)	3.48 (0.70)	3.18 (0.82)	3.33 (0.72)	3.52 (1.01)	3.46 (0.71)	3.24 (0.80)	3.24 (0.77)	3.71 (0.99)	3.38 (0.78)

Abbreviations: BMI = body‐mass‐index; EE = emotional eating; EXE = external eating; RE = restraint eating.

### 3.1 BMI

**FIGURE 1:**
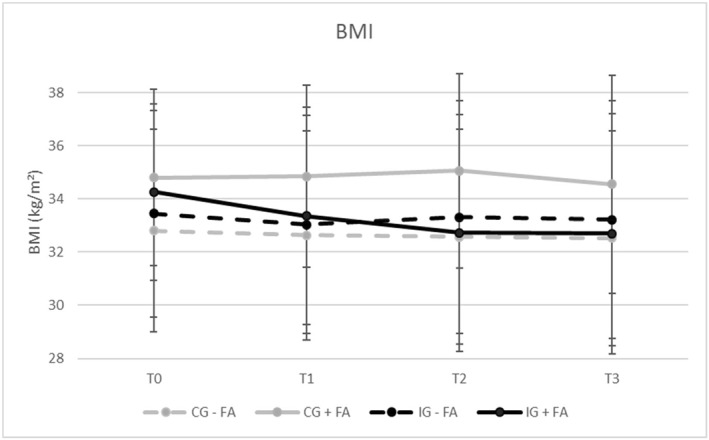
BMI progress over time (T0–T3) separated for groups.

Overall, analysis of variances revealed a decrease of **BMI** over time, *F*(2.20, 460.1) = 5.05, *p* = 0.002, *ηp* ^2^ = 0.024, as well as a significant interaction of **BMI × group × FA**, *F*(2.20, 460.1) = 3.54, *p* = 0.026, *ηp* ^2^ = 0.017 (Figure 1). Within the **IG − FA**, Bonferroni‐adjusted post hoc tests revealed a significant (*p* = 0.008) decrease in BMI between the baseline (T0) and the posttreatment assessment (T1) (*M*_Diff_ = −0.41, 95%‐CI [−0.08, −0.75]). Within the **IG + FA**, Bonferroni‐adjusted post hoc tests also revealed a significant (*p* = 0.011) decrease in BMI between T0 and T1 (*M*_Diff_ = −0.90, 95%‐CI [−0.14, −0.1.66]). Moreover, BMI in the **IG + FA** decreased significantly (*p* < 0.001) between T0 and T2 (*M*_Diff_ = −1.53, 95%‐CI [−0.52, −2.55]) and (*p* = 0.006) between T0 and T3 (*M*_Diff_ = −1.57, 95%‐CI [−0.31, −2.83]). At T0, the BMI in the **IG + FA** (*M* = 34.26 kg/m^2^, SD = 3.32 kg/m^2^) was higher on a descriptive level compared to the **IG − FA** (*M* = 33.44 kg/m^2^, SD = 3.88 kg/m^2^). Yet until T3, the mean BMI in the **IG + FA** (*M* = 32.69 kg/m^2^, SD = 4.51 kg/m^2^) decreased below the mean value of the **IG − FA** (*M* = 33.22 kg/m^2^, SD = 4.46 kg/m^2^). Until T3, participants of the **IG − FA** lost 0.70% of their initial body weight, whereas participants of the **IG + FA** lost 4.72%. The mixed model analysis supported the prior results and revealed a significant interaction of time × group × FA, indicating that FA predicted BMI progress in the IG (*ß* = 0.49, SE = 0.14, *t* = 3.61, *p* < 0.001).

### 3.2 Eating Styles

#### 3.2.1 Emotional Eating

**FIGURE 2:**
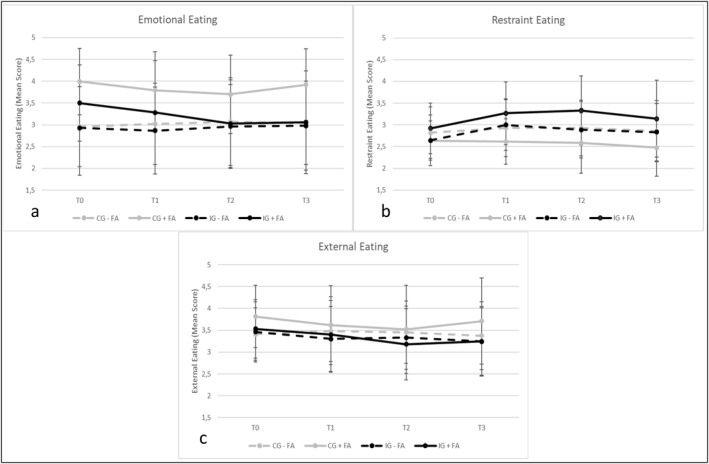
Progress in eating styles over time (T0–T3) separated for groups ((a) emotional eating, (b) restraint eating, (c) external eating).

Analysis of variances revealed a significant interaction effect of **EE × FA** over time, *F*(2.85, 595.37) = 5.47, *p* = 0.001, *ηp* ^2^ = 0.026 (Figure 2a). Within the **IG + FA**, Bonferroni‐adjusted post hoc tests revealed a significant (*p* = 0.011) decrease in EE between T0 and T2 (*M*_Diff_ = −0.47, 95%‐CI [−0.07, −0.87]). Moreover, EE also decreased significantly (*p* = 0.021) between T0 and T3 (*M*_Diff_ = −0.44, 95%‐CI [−0.04, −0.84]).

In addition, significant effects between group (**IG vs. CG**), *F*(1, 209) = 4.13, *p* = 0.043, *ηp* ^2^ = 0.019 and FA (**+FA vs. −FA**) were found, *F*(1, 209) = 9.65, *p* = 0.002, *ηp* ^2^ = 0.044. At T0, post hoc tests revealed significant (*p* = 0.022) differences in EE between **IG + FA** and **IG − FA** (*M*_Diff_ = −0.57, 95%‐CI [−0.08, −1.06]), as well as significant (*p* < 0.001) differences in EE between **CG + FA** and **CG − FA** (*M*_Diff_ = −1.0, 95%‐CI [−0.45, −1.61]) with higher scores for **IG + FA** and **CG + FA**, respectively. In the CG these differences remained significant at T1 (*p* = 0.009) (*M*_Diff_ = −0.77, 95%‐CI [−0.19, −1.35]), T2 (*p* = 0.033) (*M*_Diff_ = −0.63, 95%‐CI [−0.05, −1.20]) and T3 (*p* = 0.003) (*M*_Diff_ = −0.88, 95%‐CI [−0.29, −1.47]), respectively. At T3, **IG + FA** and **CG + FA** significantly (*p* = 0.017) differed in EE (*M*_Diff_ = −0.87, 95%‐CI [−0.16, −1.58]), with higher scores in the **CG + FA**.

#### 3.2.2 Restraint Eating

Analyses of variances revealed a significant increase of **RE** over time, *F*(3, 627) = 7.51, *p* < 0.001, *ηp* ^2^ = 0.035, as well as an interaction effect between **RE × group** over time, *F*(3, 627) = 4.75, *p* = 0.003, *ηp* ^2^ = 0.022 (Figure 2b).

Within the **IG + FA**, Bonferroni‐adjusted post hoc tests revealed a significant (*p* = 0.009) increase in RE between T0 and T1 (*M*_Diff_ = 0.35, 95%‐CI [0.06, 0.64]). Moreover, RE also increased significantly (*p* = 0.002) between T0 and T2 (*M*_Diff_ = 0.41, 95%‐CI [0.11, 0.71]). Within the **IG − FA**, Bonferroni‐adjusted post hoc tests revealed a significant (*p* < 0.001) increase in RE between T0 and T1 (*M*_Diff_ = 0.36, 95%‐CI [0.23, 0.49]), as well as between T0 and T2 (*M*_Diff_ = 0.25, 95%‐CI [0.11, 0.38]) and (*p* = 0.004) between T0 and T3 (*M*_Diff_ = 0.19, 95%‐CI [0.05, 0.33]). Yet, between T1 and T3 RE significantly (*p* = 0.007) decreased again (*M*_Diff_ = −0.17, 95%‐CI [−0.03, −0.31]).

#### 3.2.3 External Eating

Analyses of variances revealed a significant decrease of **EXE** over time, *F*(2.82, 589.63) = 6.45, *p* < 0.001, *ηp* ^2^ = 0.030, as well as an interaction effect between **EXE × FA** over time, *F*(2.82, 589.63) = 3.29, *p* = 0.023, *ηp* ^2^ = 0.015 (Figure 2c).

Bonferroni‐adjusted post hoc tests revealed a significant (*p* = 0.010) decrease of EXE in the **IG − FA** between T0 and T1 (*M*_Diff_ = −0.16, 95%‐CI [−0.03, −0.29]), as well as (*p* < 0.001) between T0 and T3 (*M*_Diff_ = −0.22, 95%‐CI [−0.08, −0.36]). Within the **IG + FA** EXE only decreased significantly (*p* = 0.011) between T0 and T2 (*M*_Diff_ = −0.35, 95%‐CI [−0.05, −0.65]).

## 4 Discussion

We analysed FA in a sample of *n* = 213 individuals with overweight and obesity without comorbid eating disorders, that underwent a 12‐weeks long tailored psychological mHealth intervention. We hypothesised, that due to the design of the intervention that addresses psychological aspects of overweight and obesity and the exclusion of eating disorders, the participants of the IG with FA (IG + FA) would reduce and sustain BMI.

Throughout the 12‐week long intervention phase, the BMI decreased in the whole IG. The BMI of individuals in the IG + FA decreased even until the 9‐ and 15‐month follow‐up assessments. At baseline assessment, the BMI in the IG + FA was higher on a descriptive level compared to the IG − FA. Yet until the 15‐month follow‐up assessment, the BMI in the IG + FA decreased even below the mean of the IG − FA. While the BMI in the IG − FA decreased by only 0.22 kg/m^2^, the BMI in the IG + FA decreased by 1.57 kg/m^2^. Participants in the IG + FA lost on average 4.72% of their initial body weight, which approximately fulfils the recommendation of 5% indicating a clinically significant weight loss (NICE‐Guideline 2014).

Our results are in contrast to former studies, that reported either no influence of FA on weight loss (Lent et al. 2014), or even worse outcome for individuals with FA (Camacho‐Barcia et al. 2021; Fielding‐Singh et al. 2019; Gordon et al. 2020). Although FA is associated with weight‐cycling (Gearhardt et al. 2014), individuals in our study lost weight even until 12‐month after completing the intervention. This might be associated with the result that the I‐GENDO app effectively reduced EE in participants with FA, since a decrease in EE is a predictor for successful weight loss (van Strien et al. 2009). At the beginning of the intervention, the participants who fulfiled the criteria for FA reported higher EE scores in both the IG and CG group. That fits the assumption that eating serves as an emotion regulation strategy in individuals with FA, who experience a shift from positive reinforcement (rewarding effect of HP foods) to negative reinforcement (relieve of negative states, e.g., stress or withdrawal) (Parylak et al. 2011). Within the CG, this difference remained stable until the 15‐month follow‐up assessment. In the IG, the differences dispersed, since only participants with FA reduced EE throughout the intervention and maintained this effect until the 15‐month follow‐up assessment. The I‐GENDO app contained a module targeting emotional competences, for example identifying emotions and warning signals and relaxation exercises (Pape et al. 2022). Given the vulnerability of individuals with FA to the reinforcing effects of HP foods, the intervention might have been particularly helpful for them.

Moreover, successful weight loss and maintenance in participants with FA might have been influenced by increased RE and decreased EXE, at least until 6 months after intervention completion (Burton et al. 2007; Neumann et al. 2018; van Strien et al. 2009). Both IG + FA and IG − FA profited from the intervention concerning RE, while the IG + FA reported greater, but less maintainable increase. As described above, individuals with FA often fail to quit consuming HP foods (Schulte et al. 2015). For especially chronically ill individuals with FA RE might be a self‐care counteraction to the long‐term consequences of overconsumption, which might explain higher RE scores in the IG + FA (Rios et al. 2023). It would therefore be reasonable to assess the onset and the degree of chronicity of FA in future studies. EXE reduced throughout the intervention in the IG − FA and slightly decreased until the 15‐month follow‐up assessment. In the IG + FA, EXE decreased until the 9‐month follow‐up assessment, but this effect did not maintain until the 15‐month follow‐up assessment. Given the definition of EXE as eating due to external cues, one would expect higher vulnerability of individuals with FA to engage in EXE. EXE as measured by the DEBQ implies a somehow unspecific vulnerability toward external food cues (e.g., ‘I eat more than usually when I see others eating’.) (Nagl et al. 2016). Yet, FA is associated with higher cue‐reactivity to HP foods, but not to minimally processed foods (Schulte et al. 2019). Results of a former study from our research group indicated that FA is associated with deficits in food‐related inhibitory control, but not with general impulsivity trait (Pape et al. 2021). It would be reasonable to further analyse the influence of FA on the efficacy of the I‐GENDO app in reducing food‐related inhibitory control, rather than EXE as a general construct.

Primary analysis showed that the I‐GENDO app was effectual in treating RE (Seiferth et al. 2023). Moreover, partially beneficial effects on weight loss and EXE were found, yet not on EE. This subgroup analysis indicates that the intervention was particularly effectual for the subgroup of individuals with FA, that is concerning EE and weight loss. That might be due to the fact, that individuals with FA suffer from high psychological distress and eating pathology (Brytek‐Matera et al. 2021) and the I‐GENDO app is a tailored mHealth intervention targeting psychological aspects of overweight and obesity (Pape et al. 2022). These findings demonstrate that tailored interventions may be one way to increase treatment success for individuals not benefitting from standard interventions. It is thus also important for future research to increase our understanding of the obesity spectrum. For example, future studies are warranted to investigate potential underlying mechanisms of FA and the precise distinction to BED, for example by using behavioural paradigms like the go‐/nogo task (Kollei et al. 2018). This will help to identify further criteria for interventions specifically tailored to individuals with FA.

The advantage of our study design is that we excluded individuals with comorbid eating disorders by a standardised two‐step procedure. Therefore, the results are not influenced by overlapping symptoms with for example BED. In addition, individuals with comorbidities that might be associated with weight status, such as Insulin‐dependent type 1 diabetes, or other SUDs, were excluded from participation. On one hand that is an advantage of the study, since confounding effects were reduced, yet on the other hand, the selected sample might not be representative. Moreover, other comorbidities that might be associated with both FA and overweight/obesity, such as attention deficit/hyperactivity disorder (ADHD), as well as the chronicity of overweight/obesity were not assessed (Brunault et al. 2019; Cortese et al. 2013). Future studies should therefore assess weight history and comorbidity status and investigate potential confounding effects on the efficacy of interventions. Another advantage of the study is, that we used a tailored mHealth intervention that allows individualisation and users feel more addressed by individualised interventions compared to those using a one‐size‐fits‐all approach (Dijkstra 2008; Ryan et al. 2019). Moreover, the 9‐ and 15‐month follow‐up assessments enabled to analyse the long‐term influence of the 12‐week long intervention on eating styles and weight loss maintenance. However, there are also some more limitations that should be acknowledged. First, since the RCT was initially designed to assess the efficacy of the intervention separated for gender, the groups in this subgroup analysis are not balanced for FA. Randomisation of future study samples should be stratified for FA in order to enhance the validity of findings. The overall dropout rate (32.9% from T0 to T4) was comparable to other studies (Lugones‐Sanchez et al. 2022). Yet, due to the unbalanced subgroups and the subsequently small sample sizes in some of the subgroups (e.g., only 5 participants in the CG with FA provided data at each of the four assessments), it was not possible to conduct a completer analysis. An advantage of the study is, that the conducted multilevel analysis can flexibly deal with missing data, enhancing the validity of our findings despite the lacking completer analysis. Nevertheless, future studies should be conducted with larger sample sizes and balanced subgroups. Moreover, the attrition, especially in the CG, might be enhanced by higher monetary compensation. Gender differences were found in the primary efficacy analysis of the I‐GENDO intervention (Seiferth et al. 2023). Yet, the small sample sizes in this subgroup analysis were not sufficient to enable further analyses on the impact of gender. It would be beneficial for future studies to address that topic, since gender differences were also found in the prevalence of FA (Pursey et al. 2014). In addition, due to the restrictions in course of the COVID‐19 pandemic, only the baseline assessment was conducted as an in‐person appointment. Therefore, at the baseline assessment both measures (weight scale and self‐reported weight) existed, while at the following assessments only self‐reported weight was assessed. Comparison between the objectively and self‐reported BMI measures at the baseline assessment did not reveal any significant differences. We therefore focused on self‐reported BMIs at each of the four assessments. However, we can not exclude that this might impact the validity of the results. Unfortunately, although studies indicate an influence of the pandemic on health behaviours, it was not possible to investigate the potential impact of different COVID‐19 restrictions on the efficacy of the intervention, due to the variant times of study enrolment and data assessment periods (Melamed et al. 2022; Robinson et al. 2021). Finally, FA was diagnosed by using a self‐report questionnaire (YFAS 2.0), which is currently the gold standard in measuring FA. Yet, future studies should develop and use objective diagnostic tools, like structured clinical interviews. Recently, the Food Addiction Symptom Inventory (FASI) was developed that should be translated and validated in German (LaFata et al. 2024). These preliminary results need support from future studies that should consider the above‐mentioned limitations.

## 5 Conclusions

Our results underline the existence of a FA subgroup in individuals with overweight and obesity, who differ in the vulnerability and treatability from those without FA. In accordance with prior studies, individuals with FA reported significantly higher EE and slightly higher BMI compared to those without FA at baseline (Parylak et al. 2011; Pursey et al. 2014). The tailored intervention effectually targeted EE in individuals with FA. The efficacy of the intervention concerning long‐term weight loss was higher for individuals with FA. These results indicate that tailored interventions could increase treatment outcomes in individuals not benefitting from one‐size‐fits all approaches. Further research is needed on underlying mechanisms of FA.

## Ethics Statement

The study was conducted in accordance with the Declaration of Helsinki, and approved by the Institutional Review Board of the Ruhr‐University Bochum (Nr. 18‐6415) and the ethical board of the University of Bamberg.

## Consent

Informed consent was obtained from all subjects involved in the study.

## Conflicts of Interest

The authors declare no conflicts of interest.

## Data Availability

Individual participant data that underlie the results reported in this article can be shared after de‐identification (text, tables, figures) by the corresponding author upon reasonable request for academic and research purposes, and subject to Data Sharing Agreements. Proposals should be directed to the corresponding author. To gain access, data requesters will need to sign a data access agreement.
